# THEM6 is a prognostic biomarker for breast cancer and is associated with immune infiltration

**DOI:** 10.1038/s41598-023-49379-5

**Published:** 2023-12-11

**Authors:** Xingjia Lu, Wenlin Chen, Gengzhou Tian, Fei Ge

**Affiliations:** 1 Department of Breast Surgery, First Affiliated Hospital of Kunming Medical University, Kunming, 650223 China; 2 Kunming Medical University, No. 1 School of Clinical Medicine, Kunming, 650223 China; 3 Key Laboratory of Animal Models and Human, Disease Mechanisms of Chinese Academy of Sciences and Yunnan Province, Kunming Institute of Zoology, Yunnan, 650223 China; 4 Third Department of Breast Surgery, The Third Affiliated Hospital of Kunming Medical University, Kunming, 650223 China

**Keywords:** Breast cancer, Cell death and immune response, Computational biology and bioinformatics

## Abstract

To characterize the implications of lipid metabolism-related gene thioesterase superfamily member 6 (THEM6) in breast cancer. Several databases including The Cancer Genome Atlas (TCGA) were utilized for our meticulous bioinformatics analysis. We further performed qRT-PCR, immunoblotting and IHC assays to validate the expression of THEM6 in various breast cancer cells and tissues. In addition, we have carried out relevant functional experiments to explore the regulatory role of THEM6 in vitro. Lipid metabolism-related genes are independent factors for overall survival. According to several databases, THEM6 was significantly more expressed in cancerous tissues of breast invasive carcinoma (BRCA) compared to its paracancerous tissues. Furthermore, THEM6 overexpression was correlated with poorer overall survival of BRCA patients, serving as a separate prognostic factor for BRCA. Biological functional analyses revealed that THEM6 was associated with tumor progression and pathogenesis. Finally, we discovered that in BRCA, THEM6 expression was linked to multiple immune cell types. qRT-PCR and Western blotting experiments demonstrated a general upregulation of THEM6 expression in breast carcinoma cells. IHC showed that THEM6 was expressed in both breast cancer tissues and para-cancer tissues, but its expression level was significantly higher in carcinoma tissues. In vitro studies indicated that THEM6 increased proliferation, invasion, and inhibited apoptosis of breast carcinoma cells, while also affecting the cell cycle and promoting cancer progression. Furthermore, THEM6 may influence macrophage recruitment and polarization in the tumor microenvironment by regulating CCL2 secretion, which in turn affects macrophage recruitment in the tumor microenvironment. Our findings indicate that the overexpression of THEM6, which is linked to the development of breast cancer, is a predictor of a poor prognosis and has an impact on the degree of immune cell infiltration. Therefore, THEM6 has the potential to be a valuable target for BRCA.

## Introduction

Breast cancer is a highly aggressive form of cancer that has a significant global impact and is a leading cause of death among women^1^. In recent years, there has been a gradual increase in the prevalence of breast cancer, with an annual growth rate of 0.5 percent. It is projected that by 2050, there will be approximately 3.2 million new cases of breast carcinoma in women worldwide^2,3^. Breast cancer is traditionally classified into five subtypes based on the expression levels of different receptors in breast tissue: luminal A, luminal B, overexpressor of ErbB2, basal-like, and triple-negative subtypes. The first two are non-invasive subtypes, while the latter two are invasive forms of breast carcinoma^4^. Unfortunately, breast cancer remains a challenging disease to treat, particularly in low-resource countries and underdeveloped regions where medical conditions and patient education are inadequate^5,6^. Current strategies for breast cancer therapy primarily focus on factors such as tumor size, pattern, metastasis, as well as the expression of hormone receptors Ki67 and HER2. Treatment options include surgery, radiotherapy, endocrine therapy, and chemotherapy, all of which can significantly delay disease progression and improve patient survival rates^7^. However, the clinical effectiveness of these therapeutic approaches has been limited, highlighting the need to explore additional molecular mechanisms involved in breast tumor regulation in order to identify new diagnostic and therapeutic targets.

Recently, dysregulated lipid metabolism has been recognized as a new indicator of tumor malignancy. Mounting clinical and laboratory studies have provided evidence suggesting that dysregulated lipid metabolism plays a crucial role in tumorigenesis, development, and therapeutic processes^8^.

The THEM family is involved in regulating intracellular lipid (FA) trafficking and has been found to impact the production and β-oxidation of FAs depending on the physiological context. THEM6 (thioesterase superfamily member 6) is a protein belonging to the THEM family, consisting of 208 amino acids. It is predicted to have a transmembrane structural domain in the N-terminal region, which controls the levels of intracellular ether lipids (ELs). THEM6 is essential for inducing the endoplasmic reticulum stress response (UPR), but its exact function remains unclear^9,10^.

Lately, THEM6 (C8orf55/DSCD75) has been found to be highly expressed in cancer cells, with its expression gradually increasing as the cancer progresses. Tissue microarray (TMA) analysis revealed a significant increase in THEM6 expression in colon tumorous tissues compared to normal tissue^11,12^. Furthermore, immunostaining for THEM6 was stronger in cancerous tissues such as the stomach and breast than in normal tissues. Previous studies have suggested that THEM6 could serve as a potential biomarker for colorectal, gastric, and breast cancers^13^. A recent study discovered that THEM6 plays a role in regulating cellular lipid metabolism and interacts with various components of the endoplasmic reticulum membrane involved in protein transport. Overexpression of THEM6 also promotes the growth and migration of prostate carcinoma. High levels of THEM6 have been associated with poor clinical prognosis and correlated with elevated levels of UPR activation. The study also demonstrated a significant association between high THEM6 levels and high Ki67 expression in two cohorts of prostate patients, indicating that THEM6 is highly expressed in highly proliferative tumors^14,15^.

Knockdown of THEM6 significantly altered the lipid composition of cancer cells. Specifically, deletion of THEM6 resulted in a consistent decrease in the levels of several ether lipids (ELs), such as ether triglycerides (ether TGs) and ether phospholipids (ether PCs). Although there has been limited research in this area, ether lipids are frequently overexpressed in tumors and promote the development of cancer cells. A study also demonstrated that knocking down THEM6 affects the synthesis of new sterols and fatty acids (FAs) in cancer cells^16^. Over the years, it has been repeatedly suggested that endogenous ether lipids (ELs) and related polyunsaturated fatty acids (PUFAs) affect ion channel opening. Increased levels of ELs in breast carcinoma cells have been shown to increase the expression of SK3, a potential potassium channel that hyperpolarizes stroma membranes and facilitates the entry of calcium ions, thus controlling the occurrence of breast and prostate carcinomas^17–19^. In vivo experiments have shown that n-6 PUFA contribute to the growth of breast, colon, and prostate cancers^20^.

Currently, the TCGA database and the GEO database are the top two bioinformatics databases that are widely used^21,22^. We have utilized these public databases for extensive genomic analyses, including expression analyses, survival analyses, and immune microenvironmental analyses^23^. The KEGG database compiles KEGG pathway maps, which are manually drawn and cover metabolic and signaling pathways, pathways involved in cellular processes and biological systems, and disrupted pathways linked to human diseases^24,25^. Therefore, we analyzed the relationship between relevant genes and some signaling pathways through the KEGG database, which played a certain auxiliary role in our research^26^.

In this study, we comprehensively analyzed genes related to lipid metabolism and identified THEM6 as a gene associated with lipid metabolism using the LASSO regression model. We further investigated the relationship between THEM6 expression, prognostic value, and immunology in breast cancer through bioinformatics. This analysis aims to provide a rationale for identifying new molecular targets for future breast cancer treatment.

## Materials and methods

### Data acquisition

The data used in this study were obtained from The Cancer Genome Atlas (https://portal.gdc.cancer.gov/), Gene Expression Omnibus (https://www.ncbi.nlm.nih.gov/geo), GeneCards database (https://www.genecards.org/), and MSigDB database (http://software.broadinstitute.org/gsea/index.jsp) for breast cancer RNAseq datasets. A total of 1101 breast samples from TCGA were retrieved, integrating 1294 genes related to lipid metabolism from the MSigDB database and GeneCards database, as well as GEO datasets GSE7904 (62 samples in total, including 43 tumors, 7 normal breast, and 12 normal organelles), GSE10810 (58 samples in total, including 31 tumors and 27 controls), and GSE65194 (155 samples in total, containing 130 breast cancer specimens, 11 normal breast tissue specimens, and 14 TNBC cell lines). We downloaded the Breast Cancer (METABRIC, Nature 2012 & Nat Commun 2016) dataset from the cBioPortal database (https://www.cbioportal.org/), which consists of 2509 primary breast tumors with 548 matched normals.

### Differences in gene expression

The TIMER database (https://cistrome.shinyapps.io/timer/) identified the expression levels of THEM6 in pan-cancer and evaluated the effects of immune infiltration. Statistical analysis was performed to detect immune cell infiltration in tumor tissues using RNA-Seq expression profiling data. Additionally, we obtained the differential expression of THEM6 in BRCA by downloading the TCGA-BRCA database from UCSC Xena and conducting statistical analysis using R software. We also selected all data in the GEO database with a combined volume of samples greater than 10 in the oncology and non-oncology groups, which were normalized and log-transformed. THEM6 expression was visualized using the ggplot2 package based on R software.

Data on TCGA, GSE7904, GSE10810, GSE65194, and lipid metabolism-related genes were collected using the Limma program. Differentially expressed genes (DEGs) were obtained by selecting log2FoldChange greater than 1 or less than − 1 and p less than 0.05. The five datasets were visualized using Venn diagrams, and the superimposed DEGs were selected as the best potential genes.

### Establishment and verify risk profiles linked to survival in breast cancer patients

RNA sequence expression mapping of BRCA and accompanying clinical data were obtained from the TCGA dataset. Then, we converted tally data to TPM and performed log2 (TPM + 1) normalization on the data, while retaining the samples with clinical information. Intergroup differences in survivorship were analyzed by the log-rank test. Lasso: was analyzed at glmnet which is an R package. Cox: prognostic models were constructed by means of multivariate cox regime analysis and analyzed at survival which is an R package: Step: data were first analyzed characterized according to multifactorial cox regression and the step function was then applied in an iterative manner. In the case of Kaplan–Meier curves, predictive modeling was performed via log-rank testing with one-variable cox proportional hazards regression. In addition, the GEO dataset with external validation for the characterization of genes associated with lipid metabolism were also performed.

### Survival analysis

We obtained RNA-seq data and corresponding clinical information for breast invasive carcinoma from the TCGA database and the cBioPortal database. We used Cox regression analysis to examine KM survival analyses to complement the above survival differences among two or more groups. We used the time-dependent ROC assay to match the predicted precision of the THEM6 genes. For KM curves, we derived p-values and hazard ratios (HRs) with 95% confidence interval (CI) by Cox regression analysis.

### Molecular correlation analysis and enrichment analysis

After conducting differential analysis using the median of THEM6, genes with FC > 1 or FC < − 1 and p < 0.05 were selected as differentially expressed genes. Subsequently, these genes were subjected to enrichment analysis, including GO functional analysis and KEGG pathway analysis (http://www.kegg.jp/kegg/kegg1.html). The Gene Ontology (GO) enrichment of different genes in the higher and lower expression groups, as well as the KEGG pathways, were analyzed using the R package ClusterProfiler (version 3.0.4). The enrichment process includes genomic enrichment analysis (GSEA) as the second stage. GSEA starts by counting a set of biologically meaningful genes, such as those in a pathway, against which the group of genes is categorized based on an enrichment score.

### Cell lines and culture conditions

The Kunming Institute of Zoology, Chinese Academy of Sciences, provided an ER+ breast cancer cell line (MCF-7), a HER2+ breast cancer cell line (SK-BR-3), triple-negative breast cancer cell lines (BT-549, HCC1937, HCC1806, and MDA-MB-231), macrophage(THP1) and nontumorigenic breast epithelial cells (MCF-10A) (Kunming, China).

All cells were grown at 37 °C in an incubator containing 5% carbon dioxide. Breast cancer cells were cultured using DMEM/F12 (Gibco, USA), 10% fetal bovine serum (Gibco) and 1% penicillin‒streptomycin solution (Gibco), while MCF-10 cells were cultured using MCF10A special medium (CM-0525). Macrophage were cultured using RPMI1640 (Gibco), 10% fetal bovine serum (Gibco) and 1% penicillin‒streptomycin solution (Gibco).

### Quantitative real-time polymerase chain reaction (qRT‒PCR)

Extraction of whole cell RNA was carried out using TRIzol (Invitrogen, USA) following the manufacturer’s guidelines. The abm 5 × All-In-One RT MasterMix kit was utilized for cDNA preparation. qRT-PCR was conducted on a real-time quantitative qPCR instrument (Bio-Rad) using a BlasTaq 2 × qPCR MasterMix kit (Abm, USA). The expression of glyceraldehyde-3-phosphate dehydrogenase (GAPDH) mRNA in cells was measured and normalized to determine the relative mRNA expression for each gene. The primer sequences used in this study (Sangon Biotech, China) are listed in Supplementary Table S1.

### Western blotting

The protein expression of THEM6 in breast cancer cells was verified by Western blotting. Protein blot analysis was performed as described^27^. Briefly, total protein was extracted using RIPA buffer (Beyotime, Shanghai, China) containing a mixture of protease and phosphatase inhibitors. The BCA Protein Assay Kit (Beyotime) was used to determine the protein concentration, following the manufacturer's instructions. Proteins were separated by SDS-PAGE, transferred to PVDF membranes (Millipore, Burlington, MA, USA), and incubated overnight at 4 °C with primary antibody. Subsequently, the membranes were incubated with mouse secondary antibody and rabbit secondary antibody (1:5000 dilution) for 1 h at room temperature. Protein staining was performed using ECL Protein Blotting Detection Reagent (Tainon, Shanghai, China). The immunoreactive bands were quantified using ImageJ (NIH, Bethesda, MD, USA).

The following antibodies purchased from Affinity Biosciences (USA) were used in this study: anti-β-actin (1:5000) and anti-THEM6 (1:500) for human proteins. Mouse secondary antibody (1:10,000) and rabbit secondary antibody (1:10,000) were purchased from Cell Signaling Technology (USA).

### Immunohistochemistry

Verification of THEM6 expression in breast cancer patient tissues by immunohistochemistry. Tissue samples were dewaxed by immersion in a dewaxing solution and 95% ethanol, followed by heating in a microwave oven for antigen recovery. After blocking with 5% goat serum, anti-THEM6 (1:200, Affinity Biosciences, USA) was used for immunohistochemical analysis. Tissue specimens were incubated with THEM6 antibody for 8 h at 4 °C and with secondary antibody for 60 min at room temperature. The immunoreactivity was observed by incubating with diaminobenzidine for 5 min. Visualize the cells with 20 × and 40 × magnification. The staining effect was observed by microscopy and counted, and the results were interpreted.

### CCK-8 assay verifies the proliferative capacity of cells

The cultured cells were collected in centrifuge tubes and counted to ensure consistent cell numbers in each well of a 96-well plate. Transfection reagents were prepared by mixing siRNA (0.5 μl/well), lipo3000 (Invigentech, USA) (0.25 μl/well), and Opti-MEM reduced serum medium (100 μl/well). The cells were divided into si-NC group, si-THEM6-1 group, and si-THEM6-2 group, with 3 replicates per group. At 0 h, 24 h, 48 h, and 72 h after transfection, 200 μl of prepared CCK8 reagent (CCK8 (MCE, USA): DMEM/F-12 = 1:9) was added to each well. The cells were placed in an incubator for 1.5 h and the absorbance at 450 nm was measured using an enzyme marker.

### Transwell assay verified the invasive ability of the cells and the recruitment of macrophages

Matrigel matrix gel (original concentration 8 mg/ml) (Corning, USA) was diluted with cell culture medium at a ratio of 1:30. Each well was filled with 100 μl of diluted Matrigel gel and incubated at 37 °C in a cell culture incubator for 2–3 h until the gel solidified. Excess Matrigel gel was aspirated. Cells transfected with siRNA for 24 h were collected and washed twice with PBS. The cells were then gently resuspended in serum-free culture medium. In the lower chamber, 700 μl of culture medium containing 20% FBS was added, and a suspension of 1 × 10^5^ cells was evenly distributed in the upper chamber. The chambers were placed in a cell culture incubator and incubated for an additional 24 h. The culture medium was aspirated, and the upper chamber was washed twice with PBS. The membrane cells in the upper chamber were gently wiped off with a cotton swab. The tissues were fixed with 4% paraformaldehyde fixative (Biosharp, China) for 20 min, followed by two washes with PBS. Staining was performed with 0.1% crystal violet (Solarbio, Beijing, China) for 30 min, followed by two washes with PBS. Finally, the chambers were placed under a microscope for photography and cell counting.

siRNA-THEM6-1/siRNA-THEM6-2 and siRNA-NC were transfected with two kinds of cells, and the cells were inoculated in the lower chamber of the Transwell and macrophage THP1 was inoculated in the upper chamber of the Transwell after 24 h. The inoculation amount of the cells varied from one cell to another, and the number of migrated and invaded cells was counted after 24 h according to the operation of the Transwell migration (without matrix gel) and invasion (with matrix gel) experiments.

### Apoptosis and cycle experiments

Cells were transfected for 24 h and subsequently cultured in serum-free medium for another 24 h. After that, the cells were collected and stained using the Annexin V-FITC Apoptosis Detection Kit (Beyotime, Shanghai, China). Apoptosis was then detected by flow cytometry within 1 h. Following the 24-h transfection period, the cells were further cultured in serum-free medium for 24 h. Subsequently, the cells were gathered, fixed with 70% ethanol for a duration of 2 to 24 h, and color-coded using the Cell Cycle Detection Kit (Beyotime, Shanghai, China). Cell cycle testing was performed by flow cytometry within 1 h.

### Detection of macrophage polarization by co-culture of breast cancer cells with macrophages

The cell supernatant was collected after transfection of cells with siRNA-THEM6 and siRNA-NC, and the supernatant was added to THP1 and co-cultured for 24–48 h, then the RNA of THP1 cells was extracted, and finally the mRNA level of CD206/IL-10 of THP1 was detected by qPCR after co-culture with breast cancer cells. The co-cultured THP1 cells were collected and centrifuged, the supernatants were removed. The collected cells were resuspended with 1 ml of 0.5% BSA/PBS solution, centrifuged at 400*g* for 3 min, supernatant removed, and repeated 2 times. Cells were resuspended with 0.5% BSA/PBS solution to a concentration of 1 × 10^6^ cells/ml and dispensed into 96-well plates with 100 μl of cell suspension per well; 100 μl of 1:10,000 diluted anti-CD206 antibody (Fine Biotech, China) solution was added to each well, and the reaction was carried out for 30 min at 2–8 °C; 400*g* centrifugation for 5 min, after supernatant removal, 200 μl of 0.5% BSA/PBS solution was added and resuspended, centrifuged to remove the supernatant, and the reaction was repeated twice; for the non-fluorescent dye direct-labeled antibody, 100 μl of 1:2000 diluted Fluorescein (FITC) AffiniPure Donkey Anti-Rabbit IgG (Jackson, China) was added and resuspended, protected from light, and the reaction was repeated twice. China) was resuspended and reacted for 30 min at 2–8 °C, protected from light; centrifuged at 400*g* for 5 min, supernatant was removed and resuspended by adding 200 μl of 0.5% BSA/PBS solution, centrifuged to remove supernatant, and repeated twice; cells were resuspended with 200 μl of 0.5% BSA/PBS solution, stored at 4 °C, and analyzed by using a flow cytometer.

### ELISA to detect CCL2 secretion level in cells

The assay was performed using Human CCL2 ELISA Kit (Sino Biological, China). The plates were washed three times by adding 300 μl of washing buffer to each well and dried. Then the standards were diluted with sample diluent to 0, 2.34, 4.69, 9.38, 18.75, 37.5, 75, 150 pg/ml, and 100 μl of standard solution or cell culture medium to be tested was added to each well, and incubated at room temperature for 2 h. The liquid was discarded, and 300 μl of washing buffer was added to each well three times, and the plate was dried. Add 100 μl of pre-formulated antibody to each well and incubate at room temperature for 1 h. Discard the liquid, add 300 μl of washing buffer to each well three times and dry the plate. Add 200 μl of pre-formulated substrate solution to each well and incubate for 20 min at room temperature, avoiding light. 50 μl of termination solution was added to each well and mixed well, and the absorbance value was measured at 450 nm using an enzyme marker within 20 min.

### Statistical analysis

All experimental data obtained in this study were statistically analyzed using SPSS 26.0 statistical software and plotted using GraphPad Prism 8. The above experiments were repeated at least three times. Survival analysis was conducted using Cox analysis, log-rank analysis, and K–M curves. Spearman's correlation was used for relevance analysis. Characterization accuracy was evaluated with ROC curves. Unless stated otherwise, bioinformatics analyses were performed with R software (4.0.3). The following p-values were used to categorize and label significant values: 0 ≤ *** < 0.001 ≤ *** < 0.01 ≤ * < 0.05. "ns" indicates non-significant. All data are expressed as means (± SD) of three independent experiments.

### Ethics approval and consent to participate

This study was approved by the Ethics Committee of the First Affiliated Hospital of Kunming Medical University. All experiments were performed according to regulations. Informed consent to participate in the study were obtained from patients.

## Results

### The expression of lipid metabolism-related genes in breast cancer

**Figure 1:**
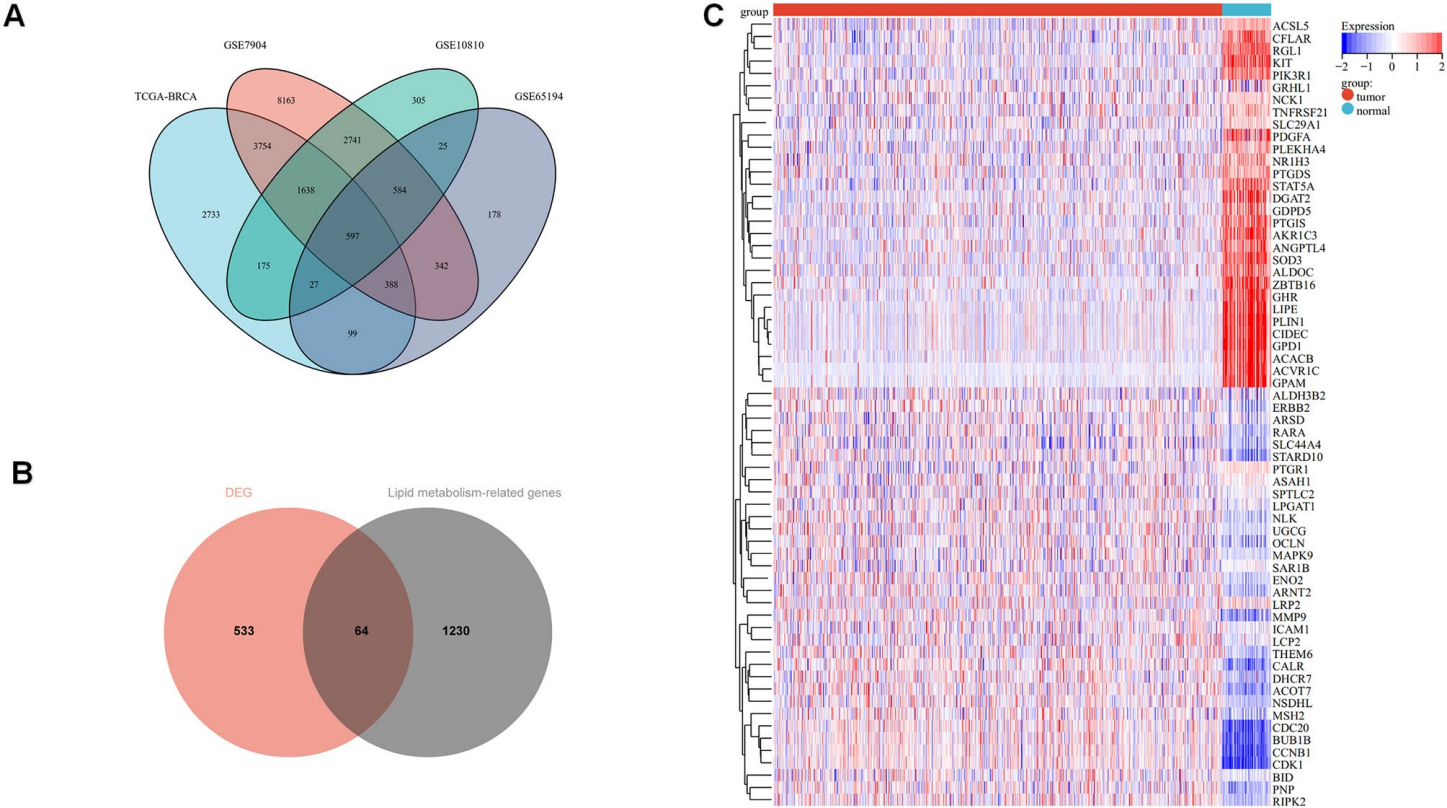
Screening for difference genes relating to lipid metabolism to construct risk signature. (**A**) Venn diagram for the crossroads of the four datasets; (**B**) Wenn diagram of the intersection of DEGs and lipid metabolism-related genes; (**C**) Heat map of lipid metabolism-related differential genes in the TCGA-BRCA cohort.

First, the limma program was used to obtain the differential genes of TCGA-BRCA, GSE7904, GSE10810, and GSE65194. The intersections of these datasets were taken to obtain the DEGs (Fig. 1A). From the MSigDB database and GeneCards database, a total of 1294 lipid metabolism-related genes were integrated. The intersection of these DEGs with lipid metabolism-related genes resulted in the identification of 64 genes (Fig. 1B). Subsequently, a heat map was constructed to display the information of these 64 genes in the TCGA-BRCA dataset (Fig. 1C) (Supplementary file S1).

### Construction of lipid metabolism-related prognostic features of breast cancer

**Figure 2:**
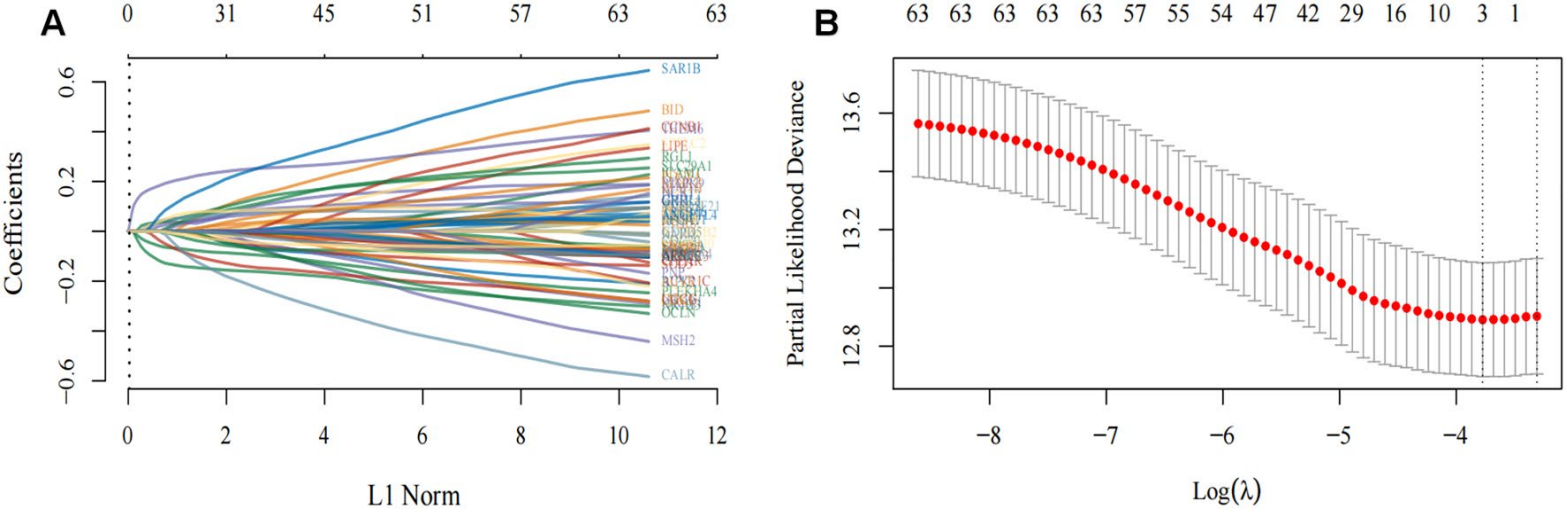
Identification of genes related to lipid metabolism in breast cancer. (**A**) LASSO lineage mapping of 64 predicted lipid metabolism-related genes. (**B**) For adjusting the cross-validation of LASSO module argument choices.

To address overfitting issues, we performed LASSO regime analysis on genes related to lipid metabolism to create hazard profiles. Eventually, three genes (NR1H3, PLEKHA4, and THEM6) were selected based on the optimal λ-value (Fig. 2A,B). Additionally, we conducted a multi-factor COX regime analysis on the risk score of these three genes using the formula: risk score = (− 0.0241) * NR1H3 + (− 0.0107) * PLEKHA4 + (0.1202) * THEM6. Subsequently, weighted risk scores were assigned to breast cancer patients, and they were divided into lower-risk and higher-risk groups (Supplementary file S2).

### Expression of validated genes in a comprehensive cohort of gene expression

**Figure 3:**
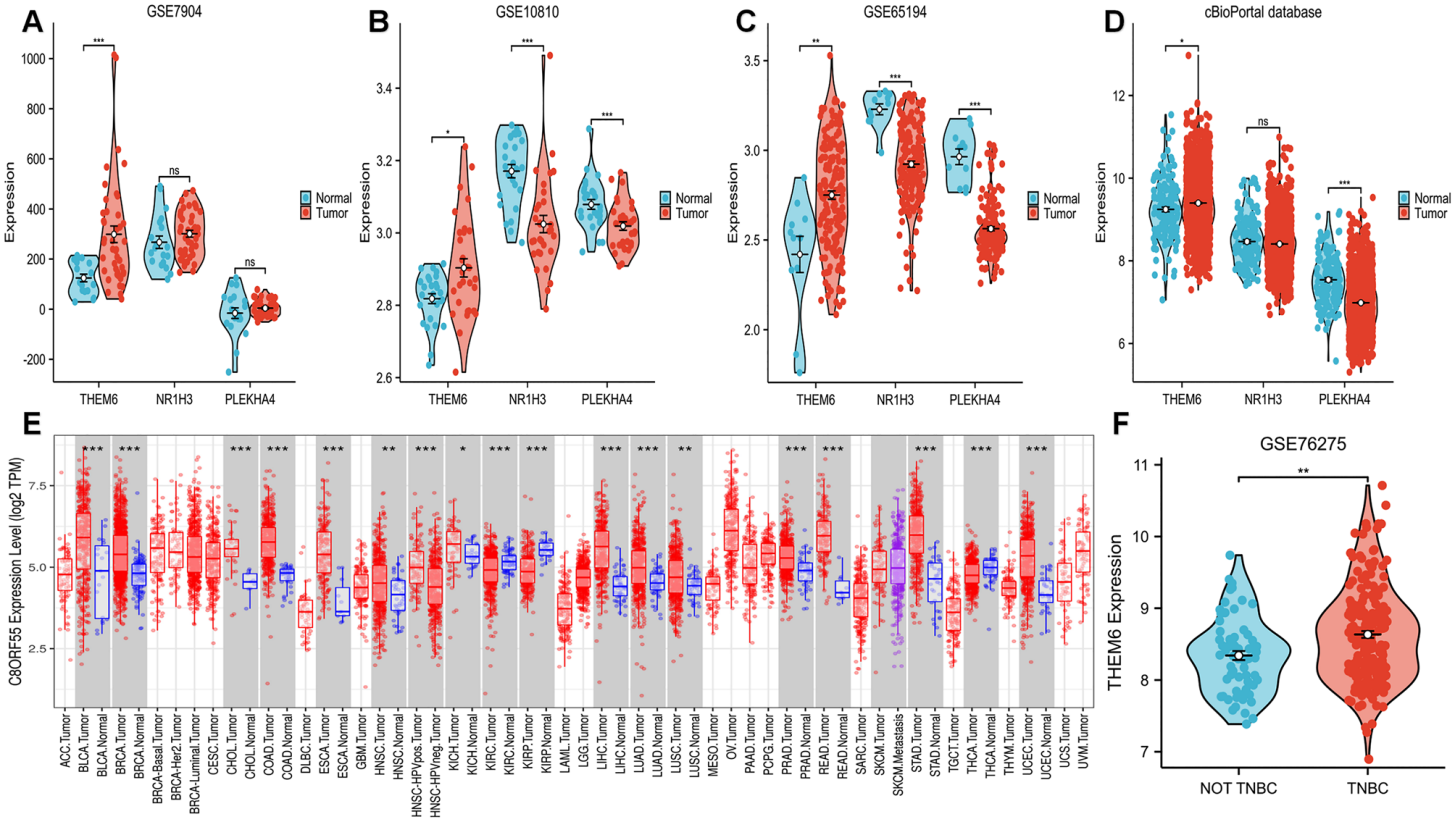
The expression levels of three genes were analyzed in the GEO dataset and cBioPortal database. In each dataset, only THEM6 was found to be upregulated in breast tumors compared to normal tissues (p ≤ 0.05). (**A**) GSE7904 (62 samples in total including 43 tumors, 7 normal breast and 12 normal organelles); (**B**) GSE10810 (58 samples in total, 31 tumors and 27 controls); (**C**) GSE65194 (155 samples in total containing 130 breast cancer specimens, 11 normal breast tissue specimens and 14 TNBC cell lines); (**D**) The cBioPortal database; (**E**) Expression levels of THEM6 in pan-cancer were analyzed using the TIMER database; (**F**) Difference in expression of THEM6 in non-triple-negative breast cancer versus triple-negative breast cancer in the GSE76275 database.

To investigate the expression of three genes, NR1H3, PLEKHA4, and THEM6, in breast carcinoma, we analyzed multiple independent cohorts, including three GEO cohorts as well as the cBioPortal database. Among these cohorts, only THEM6 showed a statistically significant difference with increased expression in carcinomas (Fig. 3A–D). To further explore the variation of THEM6 in different tumors, we also examined its expression levels in various cancer types using the TCGA database in TIMER. The results revealed a significant elevation of THEM6 expression levels in several carcinoma types, including breast carcinoma (BRCA), cholangiocarcinoma (CHOL), and paraneoplastic tissues (Fig. 3E). Interestingly, in the GSE76275 dataset, which consisted of 67 non-triple-negative breast cancer samples and 198 triple-negative breast cancer samples, we observed higher expression of this gene in triple-negative breast carcinomas compared to non-triple-negative breast carcinomas (Fig. 3F).

### Prognosis of THEM6, PLEKHA4 and NR1H3 in BRCA and its diagnostic value

**Figure 4:**
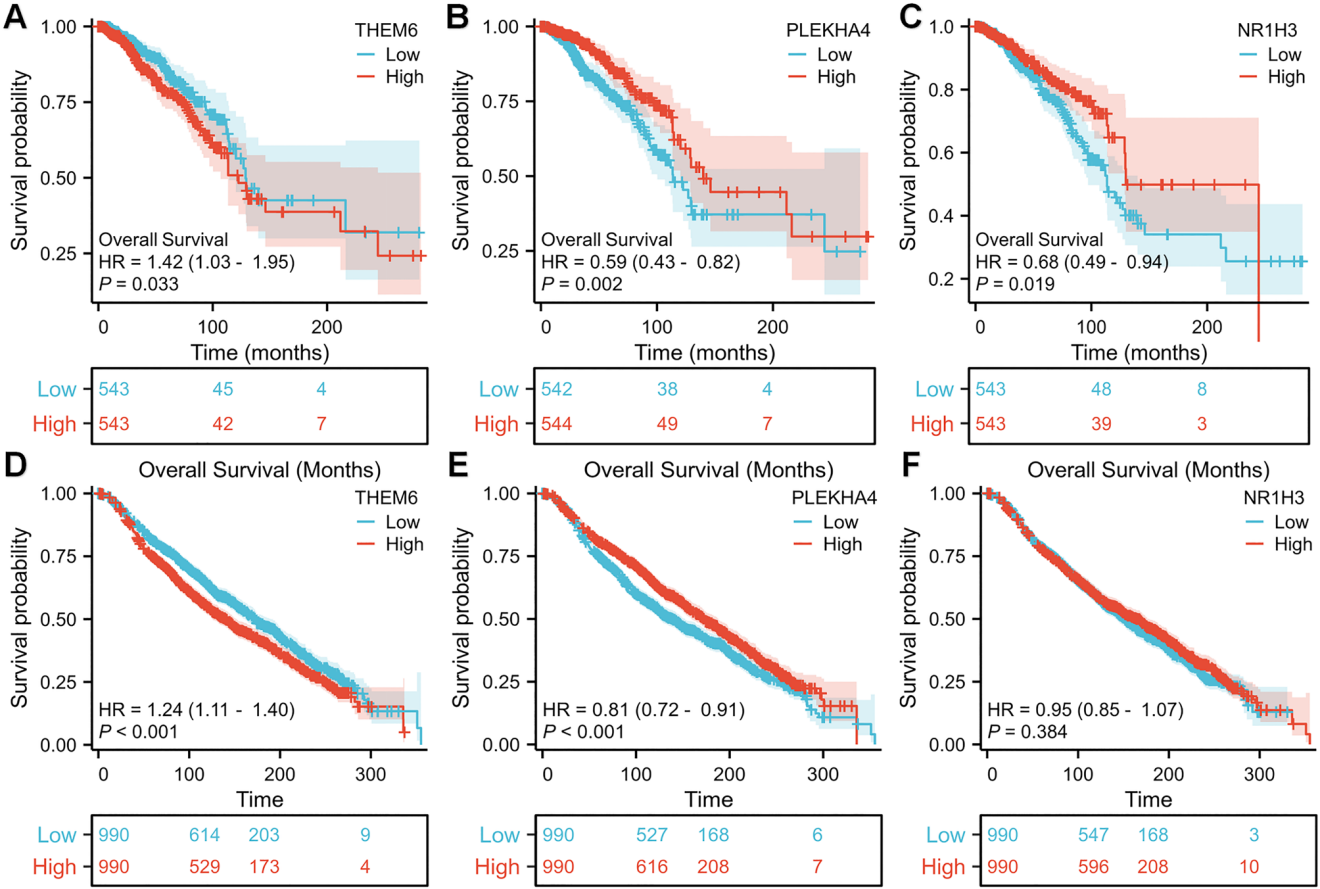
Survival prognosis based on THEM6 was analyzed by survival curve. Cox regression analysis was performed to assess the impact of (**A**) THEM6, (**B**) PLEKHA4, and (**C**) NR1H3 on overall survival (OS) in the TCGA-BRCA dataset. Additionally, Cox regression analysis was conducted to evaluate the association between (**D**) THEM6, (**E**) PLEKHA4, and (**F**) NR1H3 expression levels and OS in the cBioPortal database.

Subsequently, we employed KM analysis to determine the significance of this risk feature in the prognosis of breast cancer patients. We observed a significant association between high expression of THEM6 and adverse prognosis in the TCGA-BRCA cohort, with a 1.42-fold increased risk of death compared to low expression of THEM6 (Fig. 3A–C). Interestingly, PLEKHA4 and NR1H3 exhibited an opposite trend. Furthermore, we validated the overall survival rates of these three genes using the cBioPortal database. Consistently, high expression of THEM6 remained significantly associated with adverse prognosis, with a 1.24-fold increased risk of death, while only PLEKHA4 demonstrated a reverse trend with statistical significance (Fig. 3D–F). These findings suggest that upregulation of THEM6 expression can predict poorer prognosis in invasive breast cancer patients and may serve as a potential prognostic indicator (Fig. 4A–F).

### Analysis of clinical information on THEM6 in BRCA

**Figure 5:**
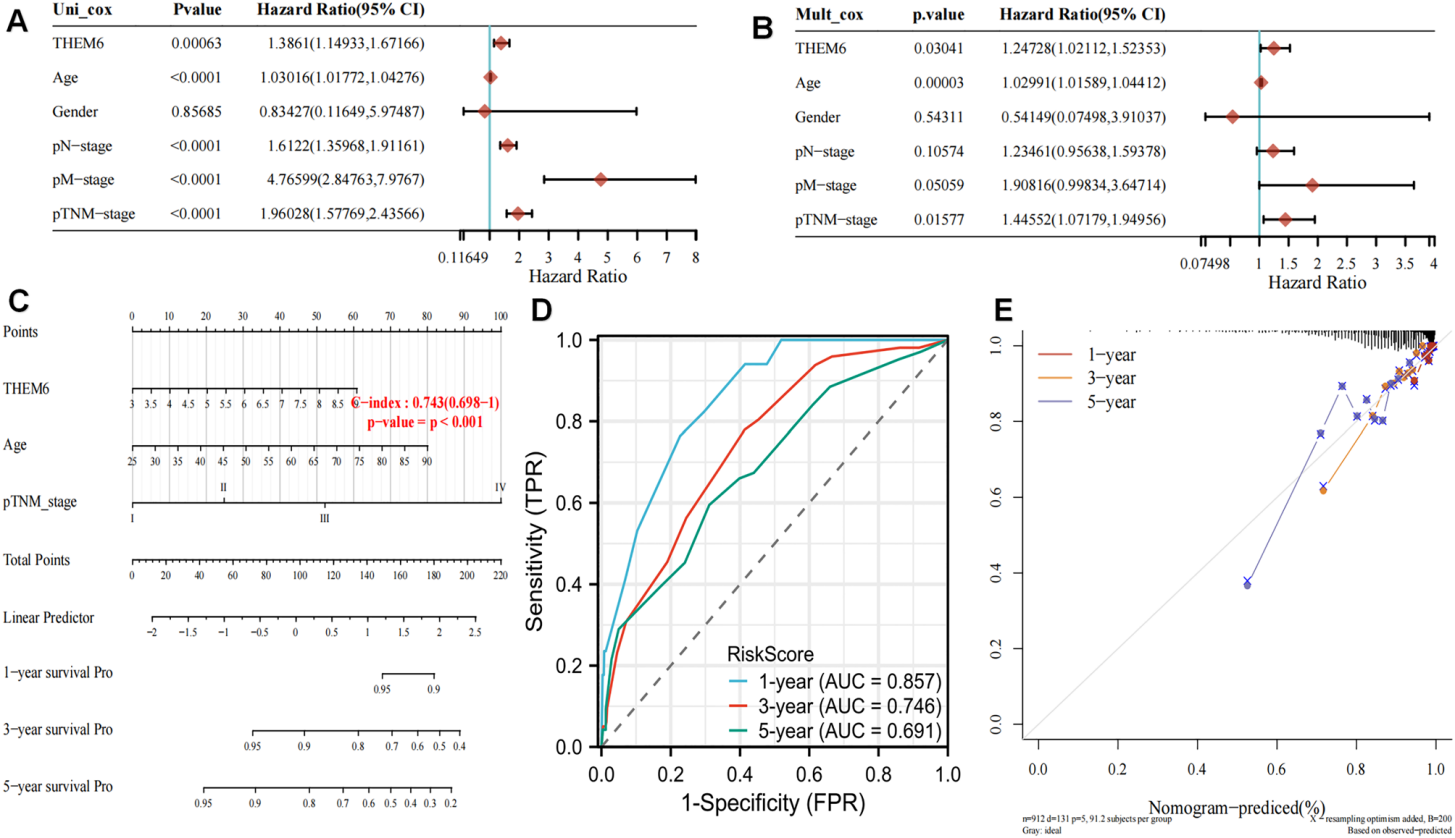
(**A**) Univariate; (**B**) multivariate Cox analysis; (**C**) column line graph predicting 1-, 3- and 5-year overall survival in breast cancer patients; (**D**) The AUC values for the time-dependent ROC curves were 0.857, 0.746 and 0.691 for 1, 3 and 5 years, respectively; (**E**) The histogram model of overall survival in this group with calibration curves for 1-, 3-, and 5-year OS showed that the survival of breast cancer patients could be accurately predicted.

We created a column line graph that integrates THEM6 expression with clinical characteristics to improve the assessment of patient survival. Additionally, we performed unifactorial and multifactorial Cox analyses of gene expression with clinical traits to analyze independent prognostic factors for this gene. The results demonstrated that THEM6 had prognostic value for age, N and M staging, and pathological TNM staging (all p < 0.0001) for overall survival (OS) in the univariate Cox analysis (Fig. 5A). However, in the multifactorial Cox analysis, only age (p < 0.0001) and pathological TNM stage (p < 0.05) maintained their prognostic value (Fig. 5B). The variables with significant differences in prognosis were used to construct a columnar line graph (nomogram) for predicting the overall recurrence rate at 1, 3, and 5 years. The nomogram had a c-index of 0.743 (p < 0.001), providing valuable guidance for clinical prognosis (Fig. 5C–E).

### THEM6 molecular correlation analysis was performed in TCGA-BRCA.

**Figure 6:**
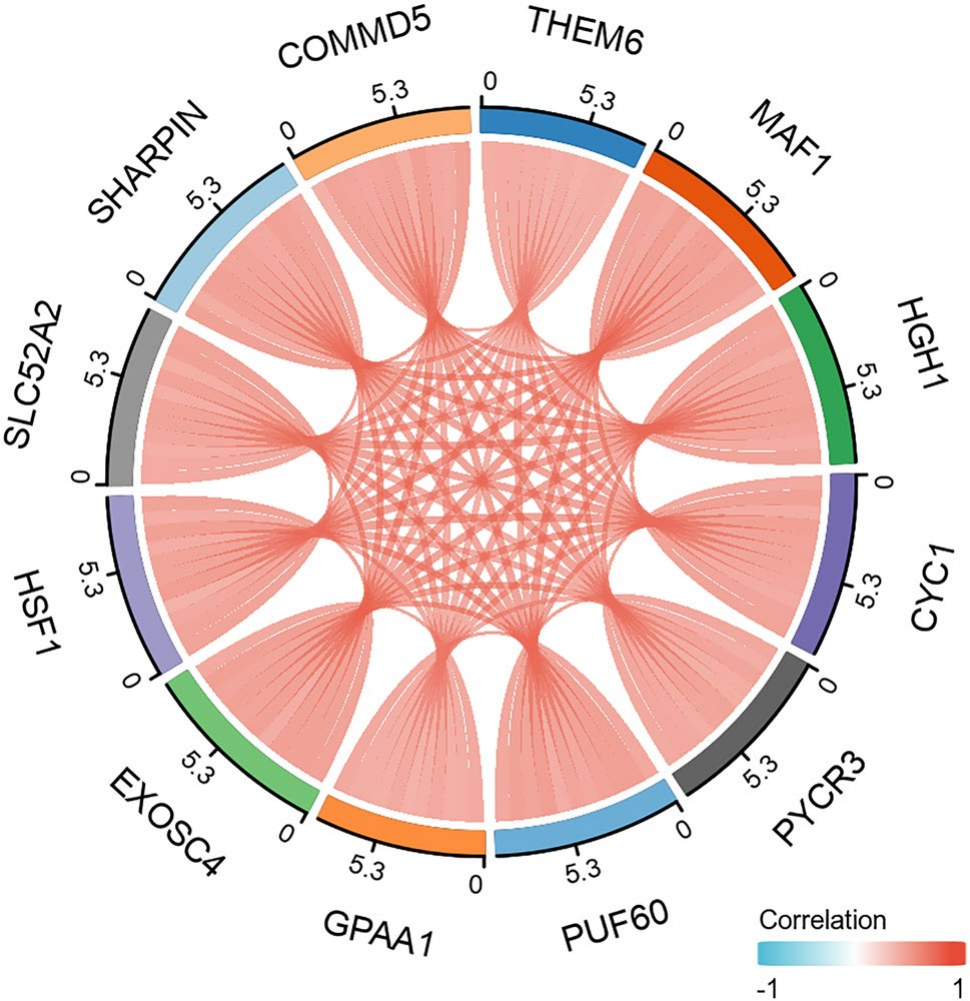
Molecular correlation analysis of THEM6 with the TCGA-BRCA data was performed and visualized to obtain chord plots.

We utilized the stat package in the R language to acquire the genes linked to the presence of the THEM6 gene (Supplementary file S3). Among these genes, 11 satisfied the threshold of |cor| > 0.7 and p < 0.05. Out of these, 11 genes were positively associated with THEM6, while none were negatively associated. To present the results, we employed the circlize package and generated a chord plot (Fig. 6).

### GO analysis and KEGG functional enrichment analysis of THEM6

**Figure 7:**
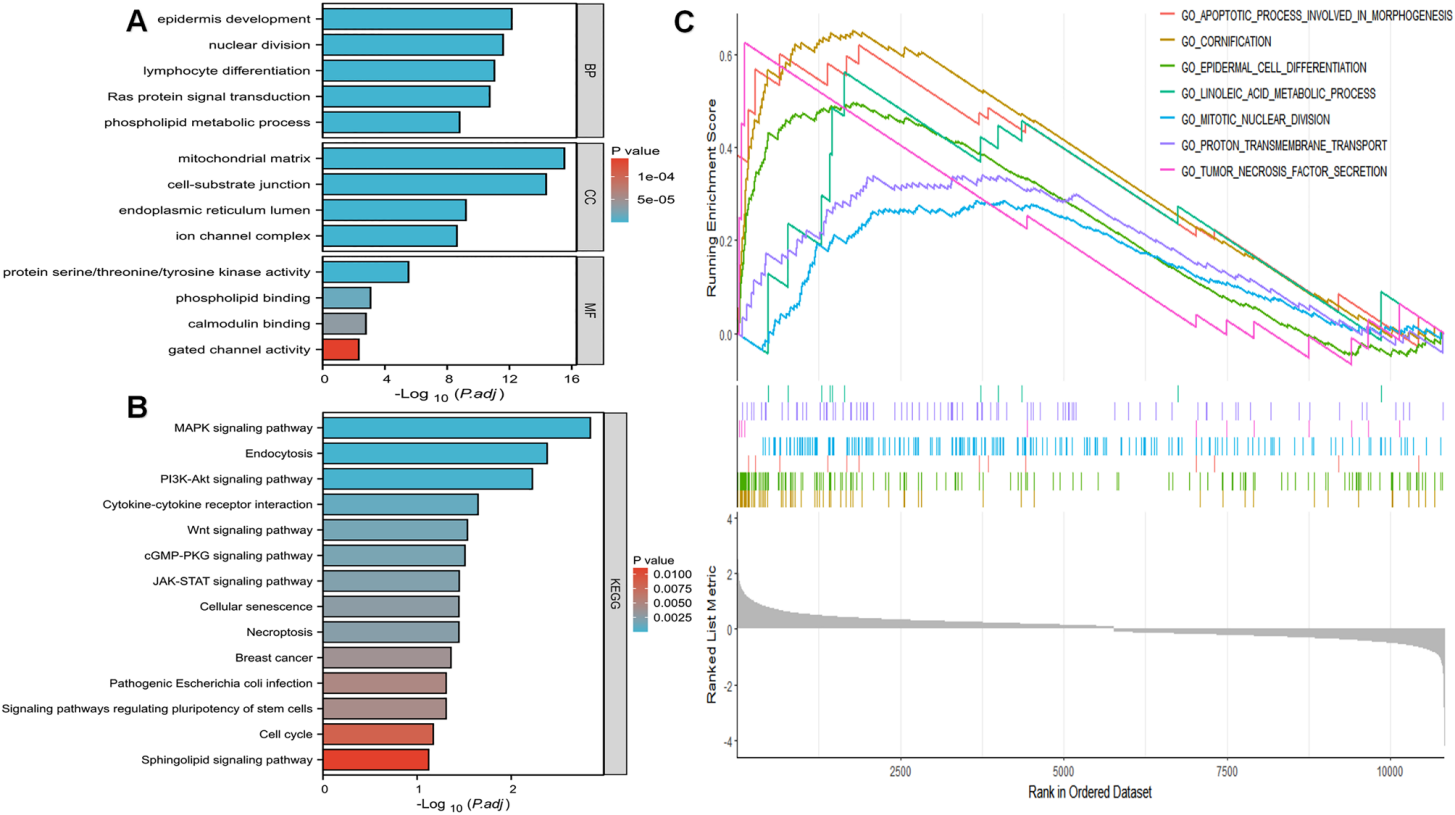
GO functional analysis, KEGG pathway analysis and GSEA analysis of THEM6. (**A**) GO functional analysis; (**B**) KEGG pathway analysis; (**C**) GSEA analysis. *BP* biological process, *CC* cellular component, *MF* molecular function.

After conducting differential analysis using the median of THEM6, genes with FC > 1 or FC < − 1 and p < 0.05 were selected as differentially expressed genes. Subsequently, these genes were subjected to enrichment analysis, including GO functional analysis and KEGG pathway analysis (Supplementary file S4). The findings indicate that the THEM6 gene may primarily be associated with epidermal growth, cell differentiation, and ion channels. These biological functions suggest a strong association with cancer development (Fig. 7A). KEGG pathway analysis revealed that THEM6 is mainly enriched in the cell cycle, PI3K-Akt signaling pathway, and MAPK signaling pathway. Additionally, it is enriched in necroptosis, breast cancer, and other diseases (Fig. 7B). GSEA demonstrated that keratinization, epidermal cell differentiation, apoptotic processes involved in morphogenesis, mitotic nuclear division, proton translocation across membranes, and linoleic acid metabolism processes were significantly enriched in the group with high-expression of THEM6 (Fig. 7C). This finding is important in highlighting the role of THEM6 in breast cancer.

### THEM6 is overexpressed in breast cancer cells as well as in tissues

**Figure 8:**
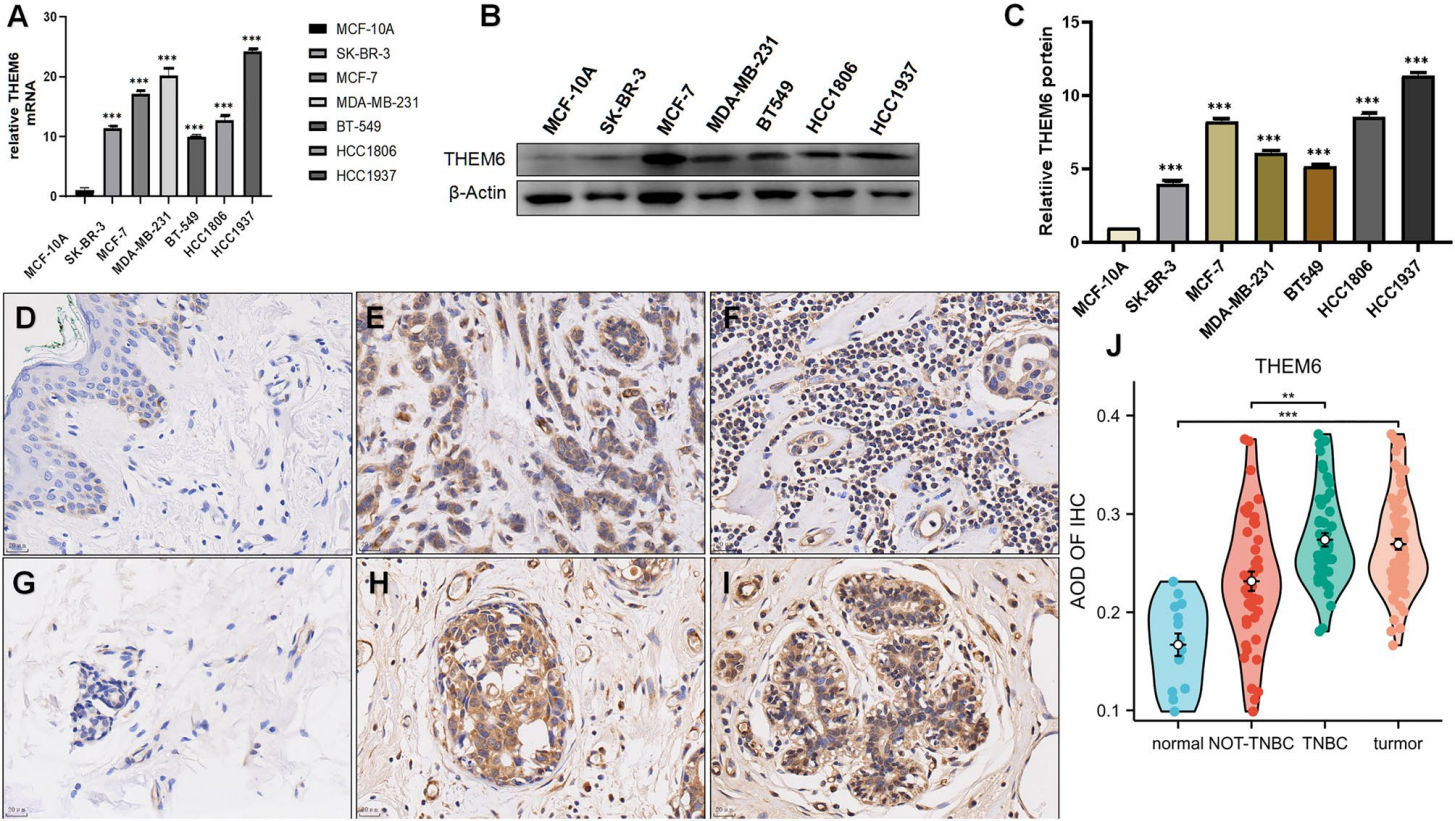
(**A**) Detection of THEM6 mRNA expression in breast cancer cell lines by q-PCR; (**B**) detection of THEM6 protein expression in breast cell lines by western blotting; (**C**) statistics of relative protein expression levels in breast cancer cell lines by ImageJ analysis of grayscale values; (**D**) breast cancer paraneoplastic tissues; (**E**) luminal A breast cancer tissues; (**F**) luminal B breast cancer tissue; (**G**) breast cancer paracancer tissue; (**H**) HER2-overexpressing breast cancer tissue; (**I**) triple-negative breast cancer tissue; (**J**) comparison of the immunohistochemistry (40 ×) results analysis of the average density of THEM6-positive cells in each type of breast cancer tissue as well as paracancer tissue (*p < 0.05, **p < 0.01, ***p < 0.001).

**Figure 9:**
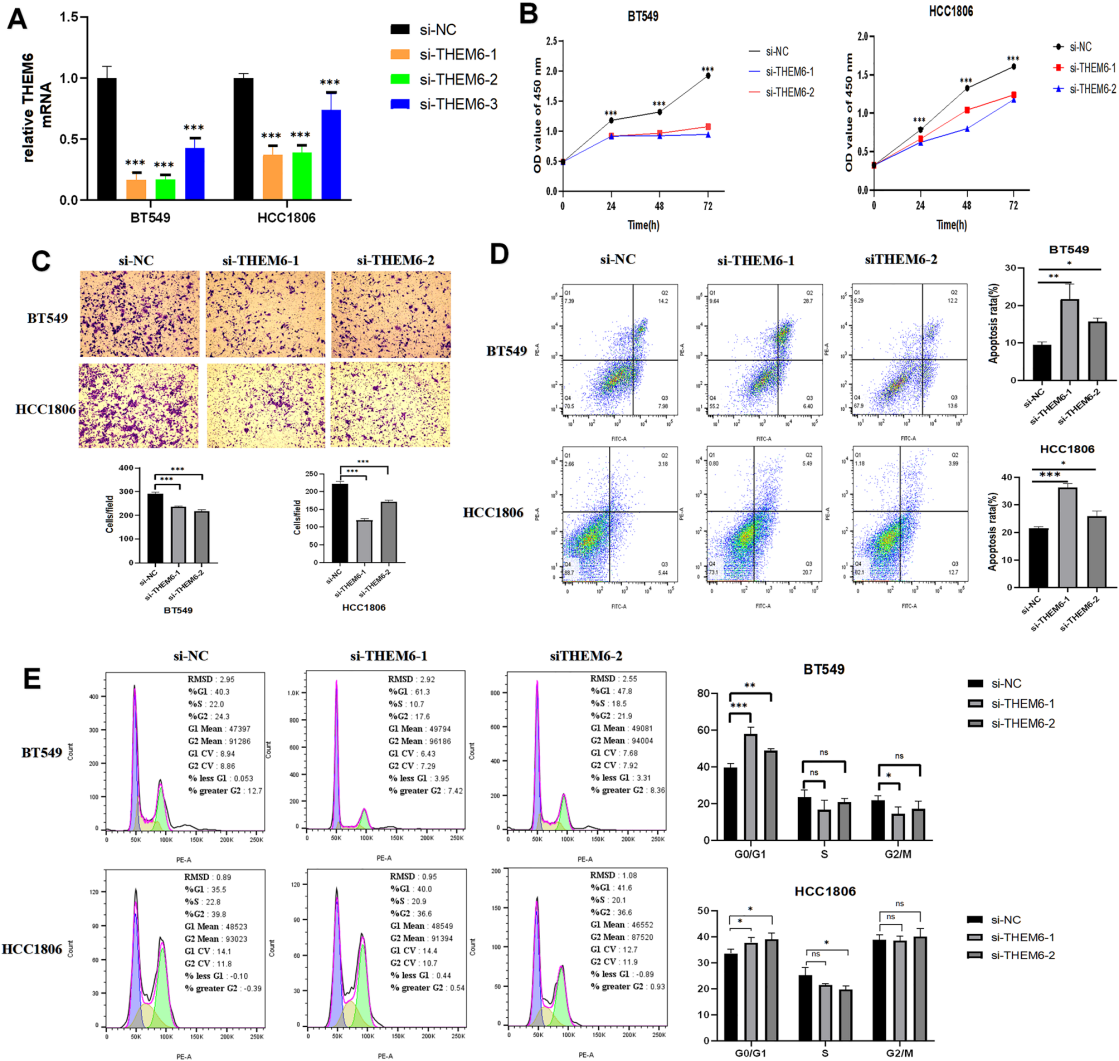
Cell-based assays were conducted to confirm the function of THEM6 in breast cancer cells. (**A**) qPCR detection of transfection efficiency in BT549 and HCC1806 cell lines in which knockdown experiments were performed. Transfection efficiency of si-THEM6-1 and si-THEM6-2 was the greatest among three siRNAs; (**B**) CCK-8 detection shows that reduced expression of THEM6 inhibits the proliferation of BT549 and HCC1806 breast cancers; (**C**) Transwell experiments revealed that BT549 and HCC1806 breast cancer cells had significantly reduced migration ability after THEM6 silencing; (**D**) Suppression of BT549 and HCC1806 cell survival by knockdown of THEM6; (**E**) Effects of silencing THEM6 followed by flow cytometry analysis of BT549 and HCC1806 cell cycles.

Relative expression levels of THEM6 were assessed in various breast neoplasm cell lines using qRT-PCR. In comparison to the triple-negative breast neoplasm cells (HCC1806, HCC1937, MDA-MB-231, and BT549), hormone receptor-positive breast cancer cells (MCF-7), and human epidermal growth factor receptor-positive cells (SK-BR-3), THEM6 mRNA expression was significantly lower in the non-tumorigenic breast epithelial cells (MCF-10A) (Fig. 8A). Additionally, western blotting revealed THEM6 protein expression in both MCF-10A and breast cancer cells (Fig. 9B). Notably, the protein level of THEM6 was higher in other breast cancer cell lines compared to MCF-10A cells, with MCF-10A serving as the control (Fig. 8C). Hence, this gene holds potential for further investigation. Immunohistochemical analysis demonstrated the expression of THEM6 in both cancerous and paraneoplastic tissues, predominantly localized in the cell membrane and extracellular matrix. Statistical analysis, employing the overall test (one-way ANOVA) and multiple hypothesis testing (Tukey HSD post hoc test), revealed significantly higher levels of THEM6 expression in cancerous tissues compared to paracancerous tissues. Furthermore, we observed that THEM6 expression was greater in triple-negative breast carcinoma tissues than in non-triple-negative breast carcinoma tissues (Fig. 8D–J). Among the 94 cases of breast clinicopathological tissues collected, 80 were malignant tumors and 14 were benign tumors. The malignant tumors consisted of 50 cases of triple-negative breast cancer and 30 cases of non-triple-negative breast cancer. Immunohistochemical staining was performed to detect THEM6 expression in the clinical histopathological sections, and the resulting staining scores were statistically analyzed alongside the corresponding clinical case data. THEM6 protein expression was found to correlate with tumor type, Ki67 expression level, and TNM stage. In addition, the expression of THEM6 increased as the malignancy of the patient's tumor increased, as indicated by the TNM stage. However, there was no significant association between THEM6 protein expression and the age of the patients, the presence of lymph node metastasis, nerve invasion, and vascular cancer thrombus (Table 1).

**Table 1:** Expression of THEM6 in breast cancer and its correlation with clinicopathological features.

Characteristic	THEM6 expression			p value
	Low (N = 47)	High (N = 47)	Total (N = 94)	
Age at surgery				1
≥ 50	27 (28.72%)	28 (29.79%)	55 (58.51%)	
< 50	20 (21.28%)	19 (20.21%)	39 (41.49%)	
Ki-67				0.01
≥ 20%	22 (23.40%)	35 (37.23%)	57 (60.64%)	
< 20%	25 (26.60%)	12 (12.77%)	37 (39.36%)	
Subtypes				0.000069
Benign tumor	14 (14.89%)	0 (0.0e + 0%)	14 (14.89%)	
Her2+	3 (3.19%)	7 (7.45%)	10 (10.64%)	
LuminalA	5 (5.32%)	5 (5.32%)	10 (10.64%)	
LuminalB	8 (8.51%)	2 (2.13%)	10 (10.64%)	
TNBC	17 (18.09%)	33 (35.11%)	50 (53.19%)	
Tumor stage				0.00015
Tis	17 (18.09%)	2 (2.13%)	19 (20.21%)	
T1	10 (10.64%)	21 (22.34%)	31 (32.98%)	
T2	19 (20.21%)	19 (20.21%)	38 (40.43%)	
T3	1 (1.06%)	5 (5.32%)	6 (6.38%)	
AJCC TNM stage				0.0014
Stage 0	17 (18.09%)	2 (2.13%)	19 (20.21%)	
Stage I	9 (9.57%)	15 (15.96%)	24 (25.53%)	
Stage II	18 (19.15%)	23 (24.47%)	41 (43.62%)	
Stage III	3 (3.19%)	7 (7.45%)	10 (10.64%)	
Neural invasion				0.71
No known involvement	44 (46.81%)	42 (44.68%)	86 (91.49%)	
Pathologically positive	3 (3.19%)	5 (5.32%)	8 (8.51%)	
Vessel invasion				0.26
No known involvement	39 (43.82%)	33 (37.08%)	72 (80.90%)	
Pathologically positive	6 (6.74%)	11 (12.36%)	17 (19.10%)	

### Knockdown of THEM6 in breast cancer cells to detect its function

We transfected three interfering RNAs into two distinct cell lines to investigate the in vitro modulatory role of THEM6 on BRCA. The qRT-PCR experiments showed that si-THEM6-1 and si-THEM6-2 had the highest transfection efficiency. Therefore, these two interfering RNAs were selected for functional experiments (Fig. 9A). CCK-8 experiments demonstrated that knockdown of THEM6 suppressed the proliferation of BT549 and HCC1806 (Fig. 9B). The results of the Transwell experiment revealed a significant reduction in the invasive ability of both breast carcinoma cells after THEM6 knockdown (Fig. 9C). Additionally, apoptosis experiments showed that knockdown of THEM6 increased the apoptosis of breast cancer cells and inhibited cell survival (Fig. 9D). Knockdown of THEM6 resulted in a prolonged G0/G1 phase in BT549 and HCC1806 cells, accompanied by a decrease in S-phase cells and insignificant changes in G2/M-phase cells (Fig. 9E). In conclusion, knockdown of THEM6 expression led to a blockage in the G0/G1 phase. Therefore, it is hypothesized that knockdown of THEM6 may impact the breast cancer cell cycle, particularly the G0/G1 phase. Thus, THEM6 appears to promote the development of breast carcinoma.

### Correlation between THEM6 expression in the tumor microenvironment and immune cell infiltration

**Figure 10:**
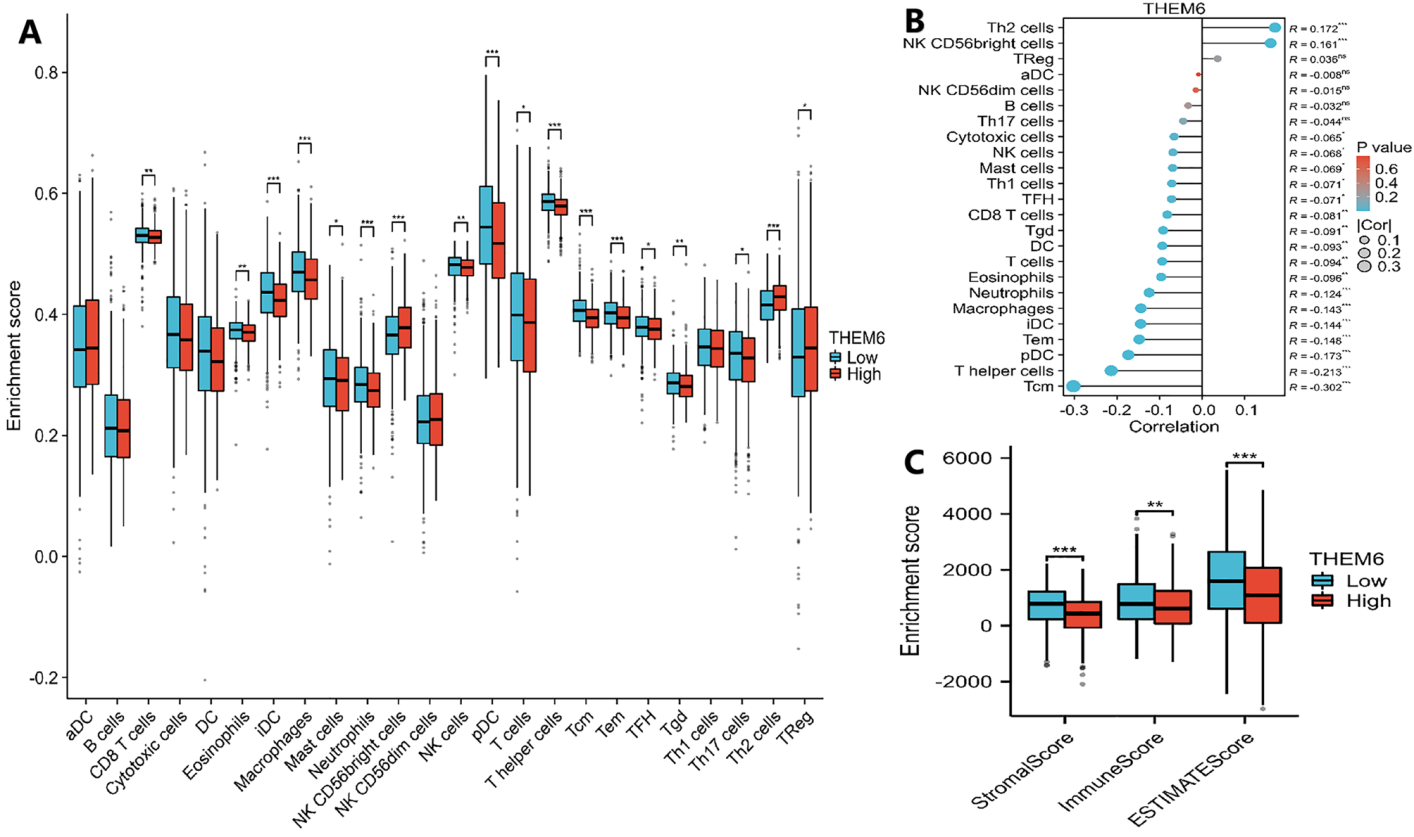
Relationship between THEM6 and immunity. (**A**) The ssGSEA algorithm initially forecasts the connection among THEM6 expression levels and immunological infiltration in per-tumor samples. (**B**) Lollipop plot showing correlation of 24 different immunocyte subtypes with THEM6 expression levels; (**C**) ESTIMATE algorithm estimated the content of immunocytes and stromal cells, and helping to prediction of immunity scoring and stromal scoring.

We analyzed the relationship between the expression level of THEM6 and the tumor immune microenvironment. In Fig. 10A, we used ssGSEA to analyze 24 immune cells from BRCA patients and found a significant correlation between THEM6 expression and the infiltration of CD8+ T cells, iDCs, macrophages, Mast cells, NK cells, and other immune cells. Additionally, we observed a negative correlation between the level of THEM6 expression and the infiltration of most immune cells (Fig. 10B). Furthermore, the high expression group exhibited lower estimated fraction, immune fraction, and stromal fraction (Fig. 10C), indicating that THEM6 has a significant impact on the immune status of the tumor microenvironment. These findings confirm our speculation that the prolongation of THEM6-associated tumors is closely linked to immune cell infiltration, which helps explain the differences in patients' survival (Table 2).

**Table 2:** Correlations between THEM6 and gene markers of immune cells in BRCA.

Gene type		Gene marker		BRCA	
		None		Purity	
		Cor	p	Cor	p
B cell	CD19	0.0058	0.86	− 0.47	***
	CD38	0.0082	0.80	− 0.41	***
CD8+ T cell	CD8A	− 0.057	0.072	− 0.51	***
	CD8B	− 0.029	0.36	− 0.48	***
Tfh	CXCR5	− 0.022	0.49	− 0.50	***
	ICOS	− 0.012	0.709	− 0.42	***
	BCL6	− 0.092	**	− 0.11	***
Th1	IL12RB2	− 0.018	0.569	− 0.23	***
	IL27RA	− 0.15	**	− 0.39	***
Th2	CCR3	− 0.17	**	− 0.26	***
	STAT6	− 0.105	***	− 0.13	*
	GATA3	− 0.015	0.63	0.26	***
Th9	TGFBR2	− 0.27	***	− 0.52	***
	IRF4	− 0.060	0.060	− 0.52	***
Th17	IL21R	− 0.083	**	− 0.47	***
	IL23R	− 0.12	***	− 0.28	***
	RORC	− 0.14	***	0.03	0.34
	STAT3	− 0.19	***	− 0.11	***
Th22	CCR10	0.021*	0.49	− 0.26	***
	AHR	− 0.18	***	− 0.24	***
Treg	FOXP3	0.0071	0.82	− 0.38	***
	CCR8	0.017	0.59	− 0.27	***
T cell exhaustion	PDCD1	0.00067	0.98	− 0.47	***
	LAG3	0.057	0.073	− 0.27	***
	CTLA4	− 0.025	0.42	− 0.42	***
Macrophage	CD68	− 0.026	0.41	− 0.35	***
	ITGAM	− 0.16	**	− 0.34	***
M1	NOS2	− 0.14	*	− 0.06	0.064
	CD86	− 0.092	**	− 0.38	***
	CD80	− 0.020	0.52	− 0.25	***
M2	ARG1	− 0.040	0.18	− 0.12	***
	CD163	− 0.022	0.48	− 0.32	***
TAM	HLA-G	− 0.0051	0.87	− 0.20	***
Monocyte	CD14	− 0.051	0.11	− 0.36	***
	FCGR3B	0.026	0.41	− 0.13	*
NK	XCL1	− 0.083	**	− 0.45	***
	NCAM1	− 0.19	***	− 0.30	***
	KIR3DL1	− 0.018	0.58	− 0.29	***
	CD7	0.0042	0.89	− 0.49	***
Neutrophil	FUT4	− 0.19	***	− 0.40	***
DC	CD1C	− 0.19	***	− 0.52	***
	THBD	− 0.26	***	− 0.31	***
	CLEC9A	− 0.20	***	− 0.48	***
Breg	CD1D	− 0.17	***	− 0.53	***
	CD5	− 0.049	0.12	− 0.53	***

**Figure 11:**
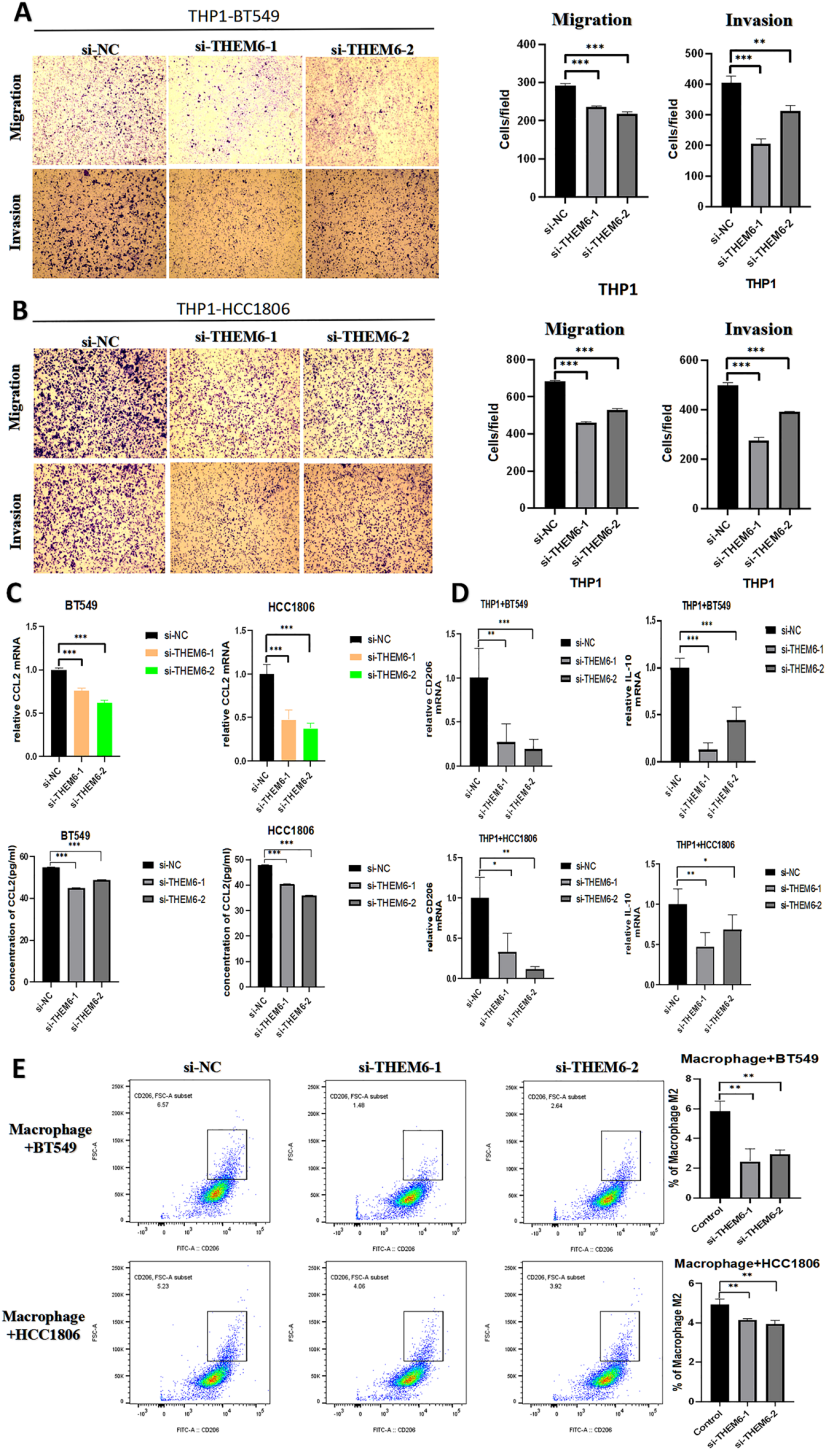
Role of THEM6 in the tumor microenvironment. (**A**) Transwell assay knockdown of THEM6 on macrophage THP1 recruitment in the TME of BT549 cells; (**B**) Transwell assay knockdown of THEM6 on macrophage THP1 recruitment in the TME of HCC1806 cells; (**C**) Detection of cellular CCL2 mRNA levels by qRT-PCR and ELISA and generated CCL2 content; (**D**) CD206 and IL-10 mRNA levels of cells with THP1 detected by qRT-PCR; (**E**) polarization of macrophage THP1 detected by flow cytometry.

After transfecting cells with siRNA-THEM6-1/siRNA-THEM6-2 and siRNA-NC for 24 h, BT549/HCC1806 cells were co-cultured with THP1 cells. The results demonstrated that knocking down the THEM6 gene inhibited the recruitment ability of triple-negative breast cancer cells to macrophage THP1 through observational counting (Fig. 11A,B). The CCL2 mRNA levels of both cells after THEM6 knockdown and their production of CCL2 by ELISA were detected using qRT-PCR, revealing that the levels of CCL2 mRNA and the production of CCL2 by the cells were reduced when THEM6 was knocked down (Fig. 11C). CD206 and IL10 are both markers of macrophage polarization towards M2 substances, and it was observed that both cells, which were knocked down for THM6 using the qRT-PCR assay, had reduced levels of both M2 polarization markers after co-culturing with THP1 (Fig. 11D). Finally, a significant reduction in the proportion of macrophages polarized toward M2 was detected using flow cytometry after co-culturing with THP1 in both cells that had THM6 knocked down (Fig. 11E).

## Discussion and conclusion

Poor prognosis, high rates of invasion and recurrence, and limited treatment options for triple negative breast cancer and advanced breast cancer have become significant contributors to breast cancer-related deaths. With the advent of precision medicine, targeted endocrine therapy for breast carcinoma has made significant advancements, leading to improved survival rates and prognoses for patients^28^.

In the current study, we examined the significance of THEM6 in breast cancer. Initially, we discovered a notable correlation between THEM6 and an unfavorable prognosis in breast carcinoma. We investigated the potential mechanism of action of THEM6 through GO enrichment analysis, KEGG pathway analysis, GSEA, and pathway correlation score. Moreover, the correlation analysis allowed us to identify genes that are significantly linked to the THEM6 gene, serving as a foundation for future investigations into the regulatory mechanism of upstream or downstream genes. Lastly, we conducted a relevant experimental study to validate our analysis.

Through the analysis of four datasets (TCGA, GSE7904, GSE10810, GSE65194) and the integration of 1294 lipid metabolism-related genes from the MSigDB database and GeneCards database, we observed a significant association between THEM6 and poor prognosis in various tumors, particularly breast cancer. Furthermore, THEM6 was found to be enriched in key pathways such as the cell cycle, PI3K-Akt signaling pathway, and calcium signaling pathway through KEGG pathway analysis. In GSEA analysis, THEM6 showed significant correlation with high expression groups involved in keratinization, epidermal cell differentiation, apoptotic process in morphogenesis, mitotic nuclear division, proton translocation across the membrane, and linoleic acid metabolic process.

Recent studies have highlighted the crucial role of immunocells in the tumor microenvironment (TME) of breast cancer. Additionally, abnormalities in lipid metabolism can greatly impact the functionality and recruitment of immunocells. However, there have been limited studies that investigate the combined effects of lipid metabolism, immune status, tumor drug resistance, and breast cancer progression. Therefore, in this study, we conducted bioinformatics analysis to explore the potential patterns of tumor immunity associated with the lipid metabolism-related gene THEM6.

In our analysis of immune infiltration, we discovered a correlation between THEM6 and the levels of six immune cell subtypes in BRCA, including CD8+ T cells, iDCs, macrophages, Mast cells, and NK cells. Our study identifies THEM6 as a potential biomarker for predicting breast carcinoma, which has important implications for assessing the progression of breast cancer and understanding the tumor microenvironment (TME). However, progress in immunotherapy for breast cancer has been relatively slow^29^. Macrophages can be classified into two categories based on their functions: classically activated macrophages (M1) that kill tumor cells, and selectively activated macrophages (M2) that promote tumor cell growth. Tumor-associated macrophages (TAMs) are a large population of macrophages found in the TME, and they primarily exhibit an M2-like phenotype. Clinical and experimental evidence suggests that TAMs promote tumor progression^30^. Research has shown that CCL2 promotes tumor progression by recruiting and activating various immune cells, while inhibiting processes such as cell apoptosis, necrosis, and autophagy to enhance cancer cell survival^31^. Furthermore, an increase in CCL2 secretion has been found to facilitate the polarization of macrophages towards the M2 phenotype, ultimately promoting tumor growth^32^. Our experimental findings demonstrate that knockdown of THEM6 leads to a corresponding decrease in CCL2 levels, along with a reduction in markers of macrophage M2 polarization (CD206/IL-10) and a decrease in the proportion of macrophage M2 polarization. Therefore, it is plausible that THEM6 may regulate the secretion of CCL2, thereby influencing the recruitment and polarization of macrophages in the TME.

However, our study has some limitations. We conducted a systematic analysis of certain functional roles of the gene using bioinformatics, but we lacked sufficient cellular functions or animal experiments to validate the results. Moving forward, we will focus on further exploring the THEM6 gene, particularly in the field of metabolomics.

**Figure 12:**
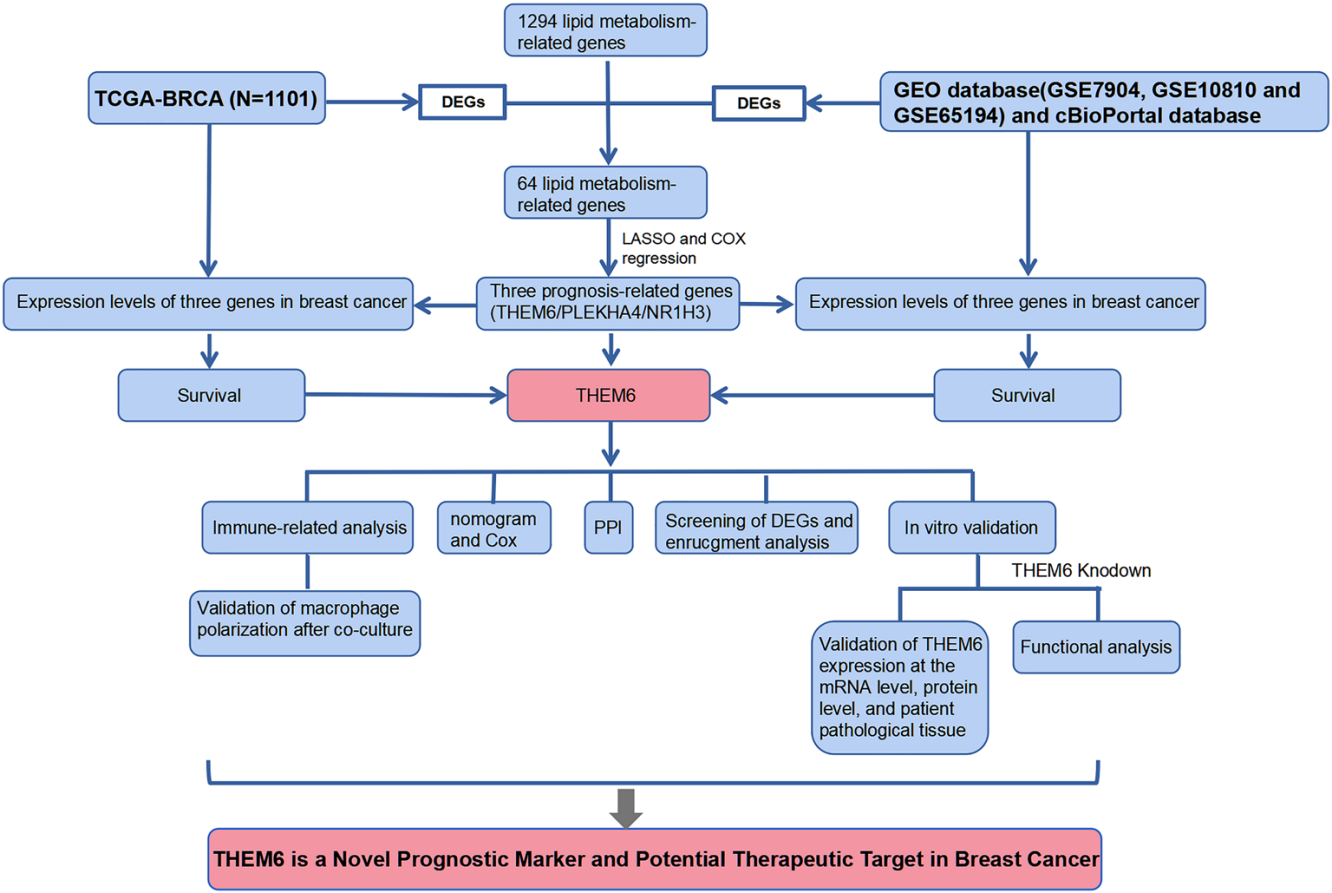
Flowchart of the THEM6 study.

In conclusion, we have established that the level of THEM6 is significantly higher in breast carcinoma tissues compared to paracancerous tissues. The elevated levels of THEM6 mRNA and protein expression are associated with a negative prognosis. Additionally, in vitro experiments have demonstrated that THEM6 promotes the proliferation and invasion of breast cancer cells, while inhibiting apoptosis. It also affects the cell cycle, particularly the G0/G1 phase, thereby promoting cancer development. Moreover, patients with high THEM6 expression levels show a correlation with Ki67 expression levels and TNM stage. Furthermore, THEM6 expression levels are positively correlated with the TNM stage of tumors, indicating that THEM6 expression increases as tumors become more malignant. Therefore, THEM6 serves as a valuable prognostic biomarker for breast cancer (Fig. 12).

### Supplementary Information

Supplementary Information 1.

Supplementary Information 2.

Supplementary Information 3.

Supplementary Information 4.

Supplementary Information 5.

Supplementary Information 6.

Supplementary Figures.

## Data Availability

Data related to this paper may be requested from the corresponding author.
