# Supplementary material for: THEM6 is a prognostic biomarker for breast cancer and is associated with immune infiltration
Source: Sci Rep. 2023 Dec 11;13:21974. doi: 10.1038/s41598-023-49379-5 (PMC10713618; doi:10.1038/s41598-023-49379-5)
Supplement: Supplementary file 6 — Supplementary Information 6. [file 41598_2023_49379_MOESM6_ESM.docx]

**Table S1:** List of primers used in this study.

| **Target Gene** | **Forward** | **Reverse** |
| --- | --- | --- |
| *mTHEM6* | gcagcactggatctcctacaacg | ggtccttggtgactcactgagc |
| *GAPDH* | aaagggtcatcatctctg | gctgttgtcatacttc |
| *Si-THEM6-1* | cagcacuggaucuccuaca | uguaggagauccagugcug |
| *Si-THEM6-2* | ggagagugggcucagugau | aucacugagcccacucucc |
| *Si-THEM6-3* | ggcucagugaugucaccaa | uuggugacaucacugagcc |
| *Si-NC* | uucuccgaacgugucacgutt | acgugacacguucggagaatt |
| *CD206* | tcagatatgccagggcgaaag | ggacatttgggttcgggagt |
| *IL-10* | ctccgagatgccttcagcag | ggcaacccaggtaacccttaaa |
| *CCL2* | ggctgagactaacccagaaacat | acttgctgctggtgattcttcta |
