# Supplementary material for: THEM6 is a prognostic biomarker for breast cancer and is associated with immune infiltration
Source: Sci Rep. 2023 Dec 11;13:21974. doi: 10.1038/s41598-023-49379-5 (PMC10713618; doi:10.1038/s41598-023-49379-5)

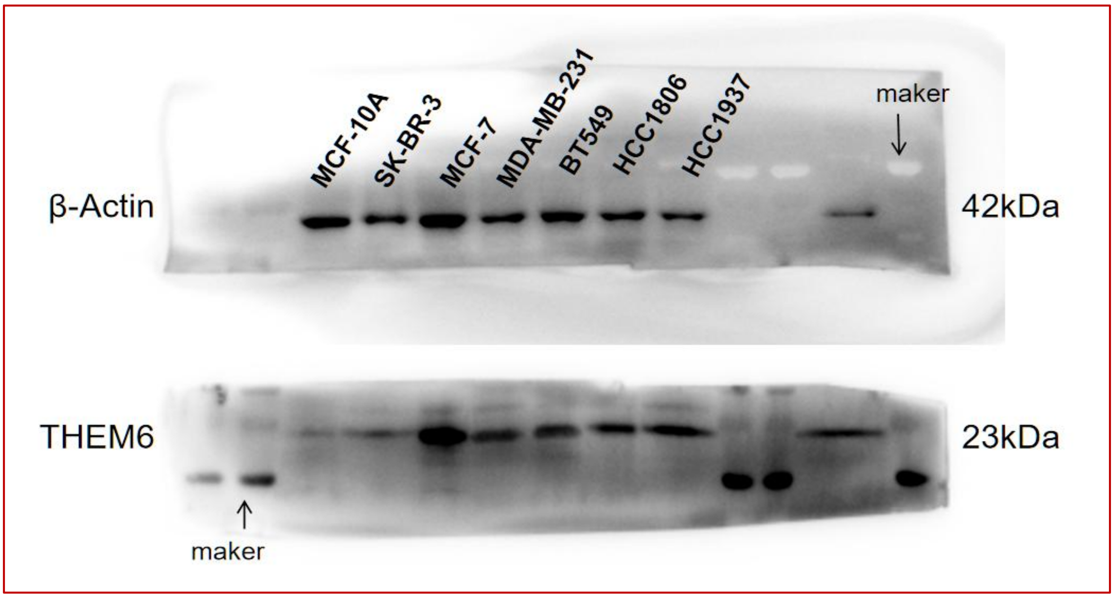

Figure 8B： We use red boxes to indicate the original images used in the article, while the ones displayed in black boxes below are the two additional repeated experiments.

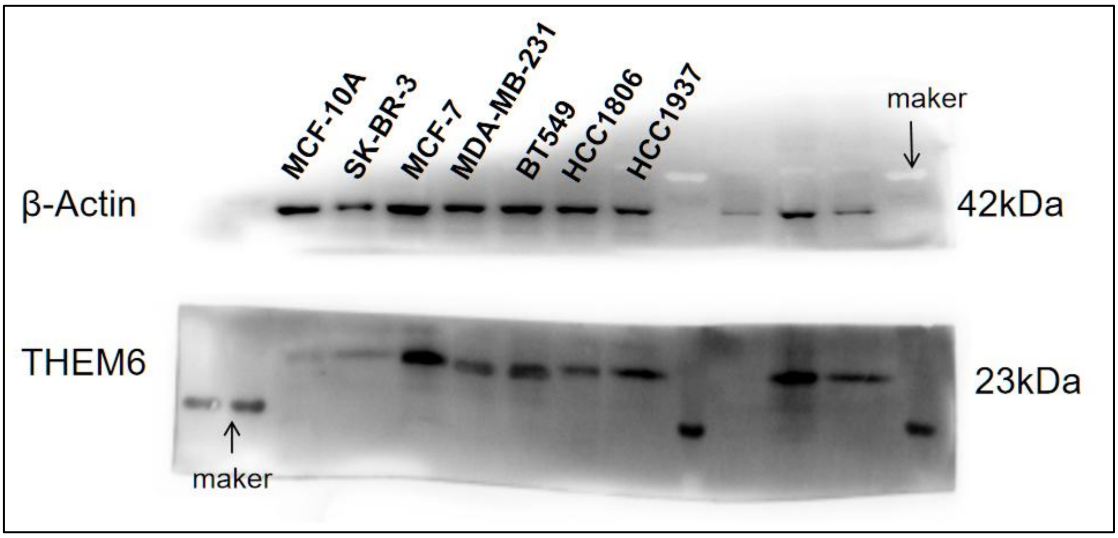

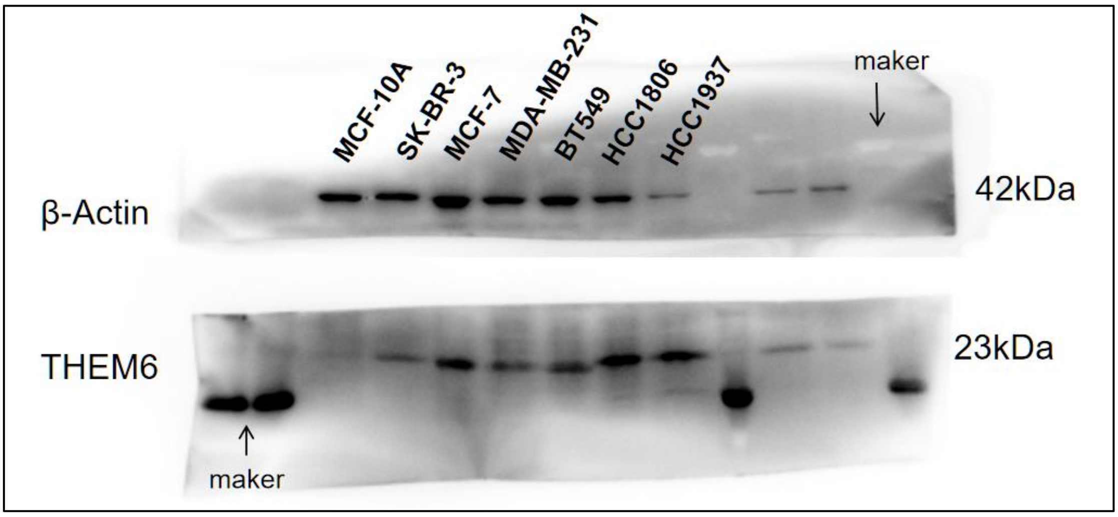

Supplement: Supplementary file 7 — Supplementary Figures. [file 41598_2023_49379_MOESM7_ESM.pdf]
